# Single-Board-Computer Clusters for Cloudlet Computing in Internet of Things

**DOI:** 10.3390/s19133026

**Published:** 2019-07-09

**Authors:** Damián Fernández-Cerero, Jorge Yago Fernández-Rodríguez, Juan A. Álvarez-García, Luis M. Soria-Morillo, Alejandro Fernández-Montes

**Affiliations:** 1 Department of Computer Languages and Systems, University of Seville, 41012 Seville, Spain; 2 School of Computing, Dublin City University, Dublin 9, Ireland; 3 Everis Spain, 28050 Madrid, Spain

**Keywords:** Internet of Things, resource efficiency, cloudlet computing, edge computing, distributed systems

## Abstract

The number of connected sensors and devices is expected to increase to billions in the near future. However, centralised cloud-computing data centres present various challenges to meet the requirements inherent to Internet of Things (IoT) workloads, such as low latency, high throughput and bandwidth constraints. Edge computing is becoming the standard computing paradigm for latency-sensitive real-time IoT workloads, since it addresses the aforementioned limitations related to centralised cloud-computing models. Such a paradigm relies on bringing computation close to the source of data, which presents serious operational challenges for large-scale cloud-computing providers. In this work, we present an architecture composed of low-cost Single-Board-Computer clusters near to data sources, and centralised cloud-computing data centres. The proposed cost-efficient model may be employed as an alternative to fog computing to meet real-time IoT workload requirements while keeping scalability. We include an extensive empirical analysis to assess the suitability of single-board-computer clusters as cost-effective edge-computing micro data centres. Additionally, we compare the proposed architecture with traditional cloudlet and cloud architectures, and evaluate them through extensive simulation. We finally show that acquisition costs can be drastically reduced while keeping performance levels in data-intensive IoT use cases.

## 1. Introduction

The rapid growth of technologies and the continuous production of data from heterogeneous sources have introduced new challenges collecting, processing and providing valuable information. Smart cities are complex systems with massive numbers of interconnected citizens, businesses, services, and utilities [1]. The interactions between Internet of Things (IoT) devices and new smart sensors are growing exponentially [2], creating massive and continuous flows of events from the most diverse origins. Furthermore, with the increase of mobile devices and the need for pervasive and ubiquitous computing, the demand for real-time processing and concurrent data access is continuously increasing [3].

Current forecasts [4] show that the large volume and latency requirements may not be met if the data generated by geographically-distributed sensors are moved to centralised data centres, due to networking limitations, since these data centres can be quite far away from the data source. Other features, such as security, privacy, and personalisation based on location could also hardly be provided to IoT workloads, such as health-care monitoring, real-time flight-support systems, and autonomous cars, if the current cloud-computing centralised architecture is not extended to provide computation closer to data sources [5]. Several computing models have been proposed to solve the aforementioned limitations. Among them, the most relevant include: (a) multi-access edge computing; (b) fog computing; and (c) cloudlet computing.

Fog computing focuses on the minimisation of latency by computing and storing temporarily latency-sensitive tasks and data in edge nodes, such as switches or servers inside the LAN network. Then, the pre-processed data and other non-critical computational-intensive tasks are sent to the cloud to be properly computed and stored. Even though such a model can meet the requirements of IoT workloads, it presents a major disadvantage to cloud-computing architectures: Computing frameworks and applications must be deployed within the local infrastructure of the final user, which needs in-house administration and maintenance. These costs might prevent small companies and entrepreneurs from implementing this model, and cloud providers from building centrally managed large-scale competitive infrastructures. On the other hand, building highly distributed large-scale cloud-computing data centres is not feasible due to high deployment, energy and maintenance costs.

Cloudlet computing brings computing clusters with good bandwidth close to the sensors and devices which are the source of data [6]. Cloudlets consist of small data centres, which are located usually few hops away from IoT sensors, and devices, and employ virtualisation, providing both low latency and management easiness. This paradigm allows data sources to offload workload from devices to such nearby tiny data centres, representing a middle-way between fog computing and cloud computing.

Technological developments allow us to build an effective data-processing clusters at an affordable price. With the emergence of distributed computing and high-throughput networks, it is possible to boost the computing power at lower costs. The emerging of single-board computers (SBC) such as the Raspberry Pi allows us to build low-cost small-scale distributed clusters for cloudlet computing. The Raspberry Pi is a tiny, cheap and powerful single-board computer. Since its appearance in 2012, it has been used in thousands of projects due to its versatility. Distributed computing is one of the latest fields in which is being used thanks to its ease of cluster assembly. This way, it is used as a testbed for evaluating solutions that could be later implemented in larger servers. Several of these boards are being used as small-scale low-cost computing clusters [7].

To solve certain problems, it is happening a paradigm shift: From costly and large to cheap and small devices, as well as from centralised to distributed solutions. Thus, despite the limited resources of SBCs, they have proven to ensure high availability performance. Moreover, those SBCs also have an extremely low power draw, which, together with their reduced price, lead to interesting cost-effective solutions.

Cloudlet computing finally enables cloud-computing providers to serve a model that could compete with fog computing, since both costs and latency are affordable for IoT clients and workloads. In our cost-effective cloudlet-computing model, Raspberry-Pi clusters are proposed as edge-computing nodes since they can bring enough computing power at low cost in most scenarios. Our contributions in this paper include:

(a) A resource-efficient architecture based on single-board computers for cloudlet computing that can bring computing power and low latency to IoT environments at a low cost.
(b) An extensive empirical and simulation analysis that shows Raspberry-Pi clusters can be suitable for realistic IoT data streams.
(c) An extensive analysis and evaluation of the proposed cost-efficient large-scale cloudlet-computing model for data and compute-intensive environments, in terms of scheduling performance and energy consumption, through realistic simulation.

The rest of this paper is organised as follows. Section 2 outlines the state of the art of the most representative edge-computing paradigms. Section 3 presents a literature analysis on the utilisation of SBCs as cluster computing nodes. Section 4 evaluates the suitability Raspberry-Pi Kafka cluster architecture and the tested configurations. The proposed cost-effective large-scale collaborative cloudlet-cloud architecture model is presented and extensively evaluated in Section 5. Finally, conclusions and future lines of work are reported in Section 6.

## 2. Edge Computing Paradigms

Data are becoming the main input for rich information-based decision making in modern societies and business [8]. Modern cities are incorporating a large number of sensors, which builds a huge IoT network. More than 50 billion smart sensors and devices are expected to be connected by 2020 [4]. The rapid growth of smartphones and the increasing number of sensors in our cities provide endless streams of events data and measurements of many parameters in real or near-real time [9]. Even if the concept of ubiquitous computing [10] is not new, technologies such as Machine to Machine (M2M), IoT, and, more globally, Internet of Everything (IoE) are fostering the development of applications that make use of real-time geographically-distributed data [11].

These new paradigms involve new challenges such as how to collect, transport, analyse, store and subsequently use this geographically-distributed flood of data with low latency, high throughput, and cost and energy efficiency. All those challenges could be seen as business opportunities for enterprises, but, to achieve this, scalable distributed stream processing systems will be required to tackle high-volume data streams in a timely and responsive fashion, as the value of particular knowledge decreases quickly with elapsed time [12]. Furthermore, concurrency, consistency, crash recovery, and synchronisation, are critical factors in massively distributed environments [13].

Some of the requirements might exceed the cloud-computing infrastructures capacities due to bandwidth and latency limitations [14]. Several computing paradigms have been proposed [15] to address the aforementioned limitations. The most relevant edge-computing models are discussed in the following sections, and a summary of our analysis is shown in Table 1.

### 2.1. Multi-Access Edge Computing

The concept of multi-access edge computing, also known as mobile edge computing (MEC), was presented by the European Telecommunications Standard Institute (ETSI) in 2014. This computing model was developed to “provide IT and cloud-computing capabilities within the radio access network (RAN) in close proximity to mobile subscribers” [16].

MEC has the aim to reduce network stress by moving computing, network control and storage resources from centralised cloud-Computing data centres to the mobile edge by pushing resources closer to the RAN in 4G and 5G [17]. MEC paradigm does not aim to replace cloud-computing paradigm, but to complement it: geographically-distributed, user-facing and latency-sensitive workloads may be deployed on MEC resources while offline computational intensive workloads may be sent to cloud-computing data centres.

Several MEC infrastructures, algorithms, and frameworks have been proposed, including: (a) distributed computation-offloading algorithms [18,19]; (b) virtualised MEC infrastructure [20,21]; and (c) providing edge networking and protection based on SDN concepts [22,23].

### 2.2. Fog Computing

Fog computing generalises the concept of MEC by employing small devices, such as commodity servers, switches, and routers, to enrich the paradigm of cloud computing by providing some computational, storage, application, and data capacities in the edge of the network, therefore closer to users [24].

OpenFog Consortium describes formally fog computing as “a horizontal system-level architecture that distributes computing, storage, control and networking functions closer to the users along a cloud-to-thing continuum”. Fog Computing is focused on environments that present several characteristics which differ from classic cloud-computing environments, such as: (a) real-time and near real-time processing engines; (b) end-users mobility; (c) data velocity and variety; (d) geographical distribution; and (e) numerous sensors and smart devices.

Fog Computing is able to outperform traditional cloud-computing architectures in the aforementioned scenarios, since large amounts of data may exceed the bandwidth capacity of centralised cloud-computing infrastructures, and fine-grained security and business logic according to location [25] might be required. Several applications and services may benefit from this advantages, such as: (a) healthcare information systems; (b) smart living systems; (c) data-intensive multimedia applications [26]; (d) industry 4.0 [27]; and (e) smart city services [28].

Fog computing requires in-LAN software and infrastructure deployment to process near real-time local data, as well as communication and integration with the centralised cloud infrastructure required to perform more intensive computation and permanent storage [29]. This new layer might bring extra costs, overhead, and complexity. These disadvantages may prevent small companies and final users from using edge-computing business models, just the opposite to the aim of cloud computing: to provide cost- and energy-efficient computing resources to final users in a transparent way.

### 2.3. Cloudlet Computing

Cloudlet computing was introduced by Satyanarayanan et al. [6] as “a trusted, resource-rich computer or cluster of computers that’s well-connected to the Internet and available for use by nearby mobile devices”. Some authors refer to cloudlet computing with the term micro data centre [30], which was introduced by Microsoft as “an extension of traditional data centres used in cloud computing”. Cloudlets represent the middle layer of a three-layer hierarchy that includes IoT sensors, cloudlet and centralised cloud-computing facilities.

Both cloudlets and micro data centres allow users to offload their jobs to a low-latency and high-bandwidth geographically-distributed tiny data centre which provides localised cloud-computing capacities, reducing operation costs [31]. Similar to edge computing [32], such miniature clouds represent edge nodes in the proximity of mobile users, which are deployed between IoT sensors and cloud-computing infrastructures, and can process computing requests of mobile devices in real-time. Cloudlet and cloud computing employ virtualisation/containerisation to provide computational resources in the form of containers and virtual machines (VMs) which are deployed on physical servers.

Cloudlet computing enables large internet service providers to compete with solutions that are not centrally managed by such providers, such as mixed infrastructures proposed by fog computing, which employ both in-LAN hardware and software deployments specifically suited for a final user needs and cloud providers. In addition, final users in cloudlet computing may enjoy the advantages provided by cloud-computing management techniques, such as on-demand pay as-you-go business models, availability, scalability and resource and energy efficiency.

Several cloudlet-based systems have been proposed, including: (a) cloudlet architectures based on maximisation of performance in mobile cloud computing scenarios [33,34]; (b) mesh cloudlet architectures, including wireless-connected mesh devices, cloudlet and cloud infrastructures [31,35]; (c) discovery and migration models for cloudlet computing [36,37]; and (d) dynamic and energy-aware cloudlet systems [38,39].

In this paper, we extend the literature by proposing an architecture model which employs SBC micro data centres in the edge of the network, feasible as a new business model for cloud providers offering a simple application programming interface (API) to final clients, allowing them to avoid the complexity of management, deployment and maintenance inherent to fog-computing infrastructures.

To show the suitability of this proposed model, we first characterise the performance and network capabilities of SBCs for stream-processing IoT workloads, and then generalise this analysis to the behaviour of a large-scale cloudlet-computing infrastructure that implements this model.

## 3. Single-Board Computers as Micro Data-Center Servers

Single-board computer clusters, such as those composed of Raspberry-Pi devices, have been utilised in several scenarios as an alternative to traditional HPC clusters. L. Miori et al. [40] showed that Raspberry clusters may be a good alternative to traditional clusters in some private cloud and edge cloud scenarios where performance is not critical. Even better results may be obtained with new-generation single-board computers along with networking and storage tuning. Similarly, the performance of big-data frameworks was analysed for a six-nodes Raspberry cluster by d’Amore et al. [41]. They showed that this solution may be a cost-effective solution for some HPC scenarios. In [42], the key factors for the great expansion of SBC systems are described: (a) Single Board Computers can run mainstream operating systems and workloads. (b) SBC clusters replicate data center features. (c) SBC clusters are a new and distinct computational deployment paradigm. (d) SBC clusters facilitate the internet of things, smart cities, and fog and edge computing. (e) SBC clusters are a game changer in pushing application logic towards the network edge.

For this reason, it makes sense to make a deep analysis about the capabilities of these systems, concretely in cloudlet computing architecture model based on single-board-computer clusters, not only from a computational point of view, but also from the prism of efficiency and energy consumption.

Tso et al. built a cloud-computing platform based on Raspberry Pi boards in order to evaluate the capacity of these SBCs to execute large-scale applications and simulations in [43]. Kruger [44] demonstrated that a cluster of Parallella nodes can compete in terms of performance with an Intel i5-3570 server. Similarly, Saffran et al. [45] analysed performance and energy consumption of a cluster composed of cost-efficient nodes with a co-processor designed specifically for HPC while executing two Big-Data data-mining algorithms. In [46], the role of SBC-based clusters in energy efficient data centers in the context of big data applications was studied. Two instances of Hadoop were deployed on Raspberry Pi and Odroid Xu-4 platforms. The experiments carried out concluded that, using popular benchmarks for CPU execution times, I/O read write, network I/O and power consumption, SBC-based clusters are energy efficient overall. Furthermore, the results show that, in all cases, SBC architectures were much cheaper than centralised one. In addition, Raspberry Pi was evaluated by Maksimović et al. [47] as an inexpensive computing node that can be successfully utilised in diverse IoT-related scenarios, which motivates this work.

The proliferation of new devices suitable for use as SBC, with the features that this entails in terms of energy consumption and performance, has made its use standardised in large scale data processing systems. Systems such as Hadoop are commonly deployed in these infrastructures and the results are good enough to be considered a completely valid alternative to traditional data centers. In [48,49], it can be observed how the number of tasks completed with this infrastructure is high (between 72% and 91% depending on the hardware setup) and, of course, with a very important reduction in power consumption. In this way, it is confirmed that, for light to middle workload applications, SBC clusters are a very cost effective and power efficient solution for deployment in hybrid cloud environments. Finally, but no less important, from the environmental viewpoint, Raspberry Pi clusters have a substantially lower energy consumption than traditional HPC clusters and it is worth the change if it meets the performance requirements [50]. This behaviour was demonstrated by Wilcox et al. in [51], where Raspberry Pi presents approximately 75% of reduction in energy consumption per MFLOPS and approximately 90% of reduction in dollar per MFLOPS compared to traditional servers. Similar results were provided by Hamilton [52] for high-scale Internet services.

In this paper, we show that a low-cost SBC cluster can be used as a part of a cost and resource-efficient cloudlet infrastructure to serve heterogeneous IoT workloads. Real-time stream processing IoT workloads backed by a Raspberry-Pi Kafka cluster are used to prove this model is a viable option in Section 4. In Section 5, we then extend the proposed scenario to generalise multiple heterogeneous workloads that may be present in edge-computing environments.

## 4. Single-Board-Computer Cluster Performance Characterisation at Application Level for IoT Real-Time Streaming Scenarios

In this Section, we employ a single-board-computer cluster: (a) to show that SBC clusters, composed of, for instance, Raspberry Pi nodes, can bring good performance levels to data-intensive, realistic IoT workloads; and (b) to gather performance insights for the simulation of large-scale SBC clusters to evaluate and compare the proposed architecture to traditional centralised cloud-computing and cloudlet-computing models.

To this aim, we extend the realistic transportation system described in a previous paper [53] to evaluate the suitability of SBCs as edge cloudlet servers. In this IoT environment, the citizens of a smart city use a mobile application designed to monitor their paths to work and back home, and receive real-time recommendations that could improve their driving efficiency, minimise the waste of fuel and reduce their stress while driving.

**Figure 1:**
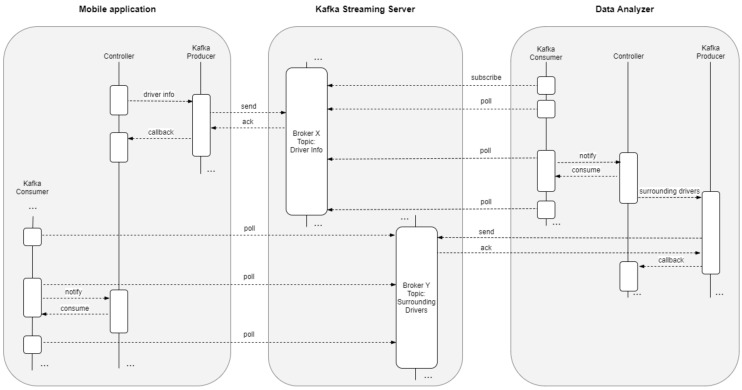
Data-flow diagram showing the interaction of the mobile application, Kafka streaming server and data-analyser module.

In this study, we simulated thousand of synthetic drivers that send their driving data to single-board-computer clusters. We tried to get real data from real users by developing an Android application, but the low number of users forced us to implement the aforementioned simulation server that mocks the very same trends of data shown by our pool of testers. Hence, it should be borne in mind that, we did not simulate the infrastructure but the data to obtain thousands of drivers. The complete data flow diagram is shown in Figure 1.

While driving, the application sends the driver’s information to the streaming server. These data, called “Vehicle Location” consist of the following information:

1. Driver unique identifier;
2. Timestamp of the sample;
3. Latitude and longitude of the vehicle in that moment;
4. Instantaneous driver stress based on the analysis of several factors, such as sudden speed variations, sharp turns, the reading of driver heart rate and previous stress values;
5. Instantaneous vehicle speed;
6. An estimation of the accuracy of the location; and
7. Extra information to make it possible to test different message sizes.

**Figure 2:**
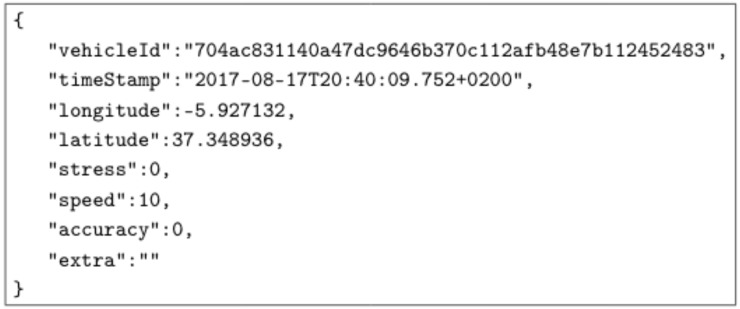
Minimum “Vehicle Location” JSON message.

This information is then encapsulated in a JavaScript Object Notation (JSON) message, resulting a minimum size of 250 bytes. An example of minimum “Vehicle Location” JSON is exposed in Figure 2:

High throughput becomes indispensable and, therefore, it is required to process a vast number of messages per second to make the recommendations to the drivers. Low latency is equally important, as the usefulness of the system relies on fast responses. An outdated message will not be useful since the driver might find himself in a completely different circumstance if the delay is too high. Consistency is also essential because the system will react one way or another depending on the driver’s history and current data.

To test the performance and the strength of the streaming cluster, a large number of people using the mobile application was necessary. The number of simultaneous application users was not achieved, and we overcame this limitation by implementing a simulator able to create thousands of simultaneous users driving along different real road paths. Each of the users generates data with the same pattern as if they were a standalone mobile application. In an attempt to be closer to reality, the simulator was implemented to generate separate threads for each simulated driver, although it involves a significant overhead regarding CPU, memory, and use of network connections. This simulator [53] is open source and it is available at https://github.com/soyjorgeyago/HermesSimulatorPro_Standalone.

There are already some spatiotemporal simulators in the literature. In [54], a very precise generator for network-based moving objects is presented in terms of road capacity and speed (external objects decrease the speed of the moving objects in their vicinity). In [55], a microscopic traffic simulation package called SUMO is described, which is aimed to help investigate urban mobility research topics. However, our intention was to generate the aforementioned business logic emulating driver’s behaviour. That is the reason none of previous frameworks were used.

### 4.1. Experimentation Setup

In this study, we analysed various criteria to characterise the behaviour of a SBC streaming cluster which uses Raspberry Pi 3 boards. Furthermore, Apache Kafka was configured to log all the events produced in order to monitor the streaming cluster during the execution of the simulators. RPi-Monitor tool, i.e., a monitoring application designed to run on Raspberry Pi nodes, was also used to keep track of resources and network throughput through an interactive Web interface to display status and graphs. This monitor caused no performance impact since the polling rate is negligible compared to the simulator utilisation.

The testbed employed in this study is composed of 16 identical standard computers that were used as clients of the streaming cluster. Each computer was equipped with an i5 2.7 GHz Dual-core processor, 8 GB of RAM using a 64 bits Ubuntu 14.04 LTS operating system and Java Runtime Environment version 8. The real-time SBC cluster utilises Kafka as stream processing engine, composed of four Raspberry Pi 3 boards: one for Apache Zookeeper and the other three as Apache Kafka nodes.

**Figure 3:**
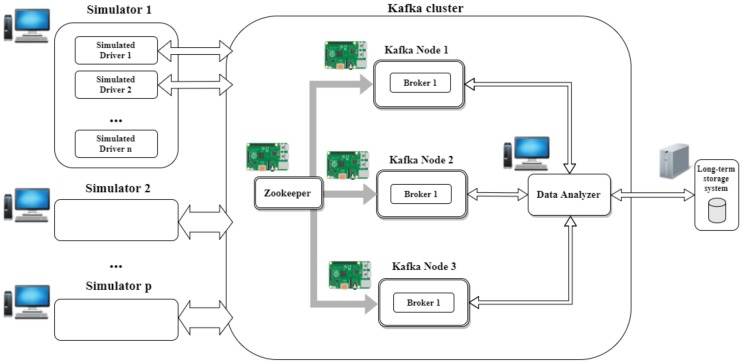
Proposed single-board-computer cloudlet micro data centre architecture. In this architecture, every node is connected to a 24-Port 10 Gigabit Switch, which acts as central node of the star topology employed. Each Kafka cluster node can see the other nodes but the intercommunications are limited to keep the cluster synchronised and Kafka producers and consumers information processing.

The architecture is based on these three main components, shown in Figure 3:

1. A messaging system to capture and publish feeds;
2. A transformation tier to manage information, enrich and deliver data; and
3. A persistence system, with tools for massive analytics.

Inside the simulator, each simulated driver created an independent Kafka producer and consumer to accurately simulate what would happen in the real world if the mobile application were used by a driver.

Furthermore, each simulator generated statistics of all simulated drivers in order to study Kafka’s behaviour for the proposed configuration. Those statistics were collected by other Kafka servers and then consumed by a Java application called Status Analyser that processes, aggregates and stores all data.

The data analyser node processed the vehicle location and data section streams, by detecting and removing outliers and transforming the information to a new data model, with the goal of sending back to the users useful information about surrounding potential risks and providing safety tips while driving. The data analyser node used high-speed data storage for caching purposes. It stored a subset of 120 MB of incoming data to prevent data loss. The results show that this cache size was enough to ensure the processing of all the information given the experimental conditions. Next, the processed information was stored in a standard massive storage to be used for offline analysis and to verify the accuracy of the suggestions and advices sent to the users.

### 4.2. Simulation Experiments

We modeled a realistic scenario where one main challenge, that is, to succeed in reaching the greater number of real-time serviced users in a cost-effective way, can be faced. In this scenario, each driver produced data every 10 s and consumed them from the streaming platform every 5 s. The messages produced started with a size of 1000 bytes and were increased in each simulation until the streaming platform could no longer cover them all.

Besides, each driver polled every 2 s the information of surrounding vehicles from the streaming server, in order to advise the driver in the case of possible hazardous situations. The average size of these data depended on the number of surrounding vehicles, being approximately 50 bytes per car. In the case of loss of connection, an in-memory storage and a retry mechanism with a configurable number of retries were implemented, to ensure no data were lost.

A series of experiments was carried out using different Kafka configurations to validate and compare the presented architectures. Each simulator collected data for seven key metrics during its execution:

1. Total number of messages generated;
2. Total number of messages sent;
3. Total number of messages received successfully by the streaming platform;
4. Total number of messages that failed to be sent;
5. Total number of messages that could be recovered;
6. Total number of messages pending to be sent; and
7. Current average drivers delay.

For our experiments, the sample rate was set up to 5 s as it is sufficient to effectively provide a proper level of detail in order to to save battery of mobile application, without overloading the systems.

For each streaming cluster configuration, the following goals were analysed:

1. The impact of the message size sent by the clients on the cluster performance.
2. The number of simultaneous drivers that could be served given a fixed message size.
3. The maximum message size that could be served given a fixed number of users.
4. The number of drivers that could be served increasing the message production rate given a fixed message size.

Ten paths were generated and used for all the simulations in order to assure that the simulation conditions were strictly identical. In addition, 1000 different driver conditions were created for each path to simulate a more realistic scenario, effectively normalising the distribution of drivers along the path and preventing all drivers sending data always at the same time. Besides, a maximum response time of 5 s was considered. The response time was measured as the time elapsed between the client sending the data and the reply being received.

The duration of experiments depended on what was being measured. In the case of finding the impact of message size, the experiments lasted 5 min each, with a pause of 2 min, during a total of 10 h, resulting 85 experiments in total. In each test, the size of the message sent was increased by 1000 bytes, starting with a 1000-byte length. Furthermore, 2000 simultaneous drivers were simulated to prevent the influence of handling a large amount of connections. On the other hand, in the case of checking the amount of simultaneous users that could be supported by the cluster, a fixed message size of 1000 bytes was set and the experiment lasted 1 h. In this scenario, the Raspberry Pi clusters were moved to an experimental room with Cat6 LAN and 16 identical computers to prove the resistance to up to 80,000 simultaneous users, starting with 10,000 simultaneous users, adding 10,000 users each time. All devices were connected to the same network following a star network topology. We used a 24-Port 10 Gigabit Switch (FS S3900-24T4S model). After analysing the evolution of the cluster’s performance, when a sharp change was noted, some short increments of 5000 users were also tested.

### 4.3. Experimentation Results

The first experiment presented in this section simulated 10 h of test conditions to evaluate how message size affects the cluster performance and behaviour, through the analysis of the slow responses rate (timeout), which was caused by a late response of more than 5 s. We employed various message sizes that were filled with unnecessary extra data, such as simulated vehicle characteristics: color, weight, and car number plate. This way, we could test how the size of the message could affect to the number of simulated cars that could be served in real time.

**Figure 4:**
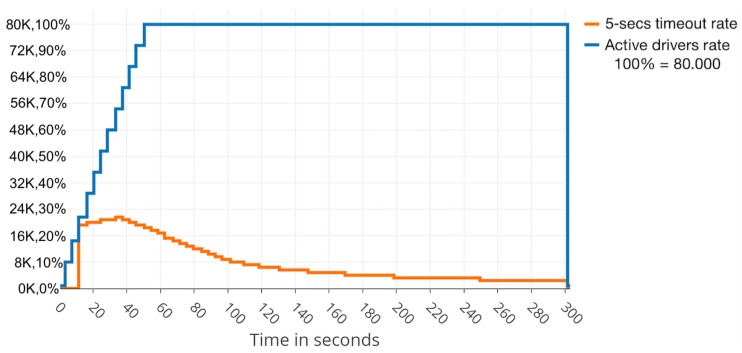
Slow responses percentage decreasing after rising to 26% with 38 KB message size.

Thanks to the load being shared among three nodes, the cluster resisted relatively well the dynamic data flow to which the cluster was exposed. Due to Zookeeper load-balancer and Kafka partitioning configuration, and the fact that cluster configuration does not use over-partitioning topics (better data balancing and consumer parallelism, but more partitions required, more open file handles and more resources needed), Zookeeper sends all the workload to one of Kafka nodes until it reaches nearly its limit. Then, it turns to another Kafka node to help the first one. It is simple to recognise when this rate limiting is happening and also simple to determine the problematic clients on the rate-limited host, as can be seen in Figure 4 close to Second 40 where the cluster recovers successfully from the sudden load of users, decreasing the slow responses rate after few seconds.

Results reveal that errors (5-s timeouts) occurred late, but there were also other implications that should be noticed, such as the inner sync traffic generated.

In the message size range of 34–36 KB, there was 14% slow responses rate that worsens gradually until the range of 46 KB where the slow responses rate was 32%, but then, curiously, it decreases a little just reaching 50 KB, being the slow responses rate of 35%.

**Figure 5:**
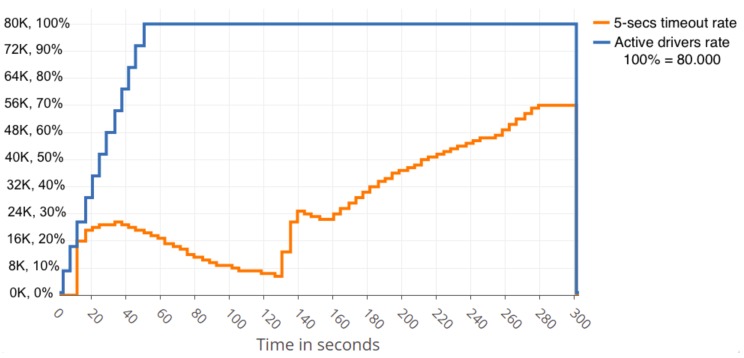
Slow responses first decreases, and then increases up to 70% with 58 KB message size.

Nevertheless, in the range of 51–60 KB, the cluster was unable to recover as with lower message sizes and the slow responses rate increased up to 70%, as can be seen in Figure 5.

After this, and despite the cluster resisting a total breakdown, the increasing of message size caused the slow responses rate in the range of 92–95% at the end of the simulations.

**Figure 6:**
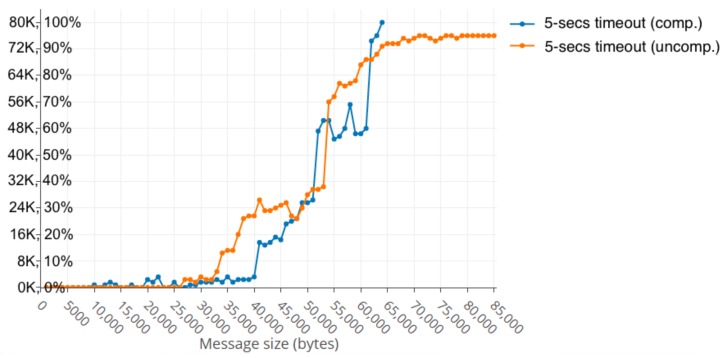
Slow responses rate without compression vs. with compression.

When using compression, the performance slightly improved, in the case of only 2000 simultaneous users, when the size of the messages was smaller than 40 KB. The first significantly slow response rate happened in the range of 41–43 KB with 17% peak and it increased to 33% when the message size was in the bounds of 50 KB. It is interesting to point out that, when using compression, the cluster broke down due to errors when the size was above 64 KB on all occasions, while, in the previous cases without compression, it resisted all simulations. Figure 6 shows that the slow responses rate occurred as the message size was increased.

**Figure 7:**
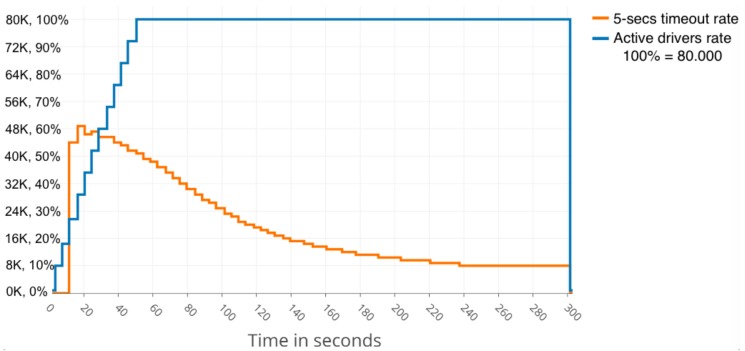
Partial recover after rising above 60% slow responses rate with a 62 KB message size and compression.

Continuing with compression, the results also reveal that, even in the range up to 62 KB, the cluster could partially recover from the load of users and, even though slow responses rate increased very quickly at first, after a few seconds, it decreased slowly, but still remained above 10% at the end, as can be seen in Figure 7.

Message sizes above 63 KB when using compression made the cluster unable to recover, even partially.

In referring to monitoring statistics, with and without compression, Table 2 summarises the results. Since the case using compression could not be completed due to breakdown of the cluster, only the common parts of the simulations were compared, that is, up to 63 KB message size. The values exposed are the averages of the measures of the three Kafka nodes during the simulations.

Results reveal that, despite the high memory usage, swap started to be used only in the latest simulations, where the message size exceeded the range of 50–54 KB. It coincided with the zone where the slow responses rate decreased more, after several increases. The cluster was responsive, even though high memory usage during compression was not used. When compression was used, the Kafka leader collapsed in two of the five 10-h simulations, when message sizes were 61 KB and 63 KB, due to its broker was getting into an unexpected error because it claimed leadership without having an in-sync replica. In the other three complete simulations, the cluster failure was due to timeouts during new leader election. This could be partially solved by making fine adjustments to timeout parameters in Zookeeper and Kafka. Finally, it is also of interest to point out that there was not a difference in the temperature when using compression.

Turning to the amount of simultaneous users that could be supported using this configuration, the experiments show that, as a general rule, the more nodes, the better fail tolerance, but there is a main drawback: it generates more sync network traffic between the nodes, also affecting the CPU and memory usage. This sync network traffic also depends on the number of connected users sending information and the health of the cluster. Thus, when one of the nodes fails, the leader election and sync tasks overload have to be tackled by the rest of the nodes that had an important pressure already. In addition, when running close to the network capacity of the cluster, the traffic overwhelms the network cards and failures and performance issues appear due to the overload. Taking this into consideration, the SBC cluster was used in the experimental room with Cat6 LAN and 16 computers to prove the resistance to up to 80,000 simultaneous users. For this series of tests, a fixed message size of 1000 bytes was used with no compression. Table 3 summarises the performance results.

The advisable maximum number of simultaneous users, under the exposed experimental conditions, stands at just under 32,000 clients. The slow responses rate would rise quickly above this reference value, although the cluster would still be operative.

#### 4.3.1. Centralised Cluster Behaviour

In this section, we use the performance results obtained in the cloud FIWARE cluster, which is deeply described in our previous work [53], to compare the performance results shown in the previous section, which were obtained using a Raspberry-Pi cluster. In [53], we considered the very same experimentation scenarios presented in the previous section of this work for results to be comparable.

**Figure 8:**
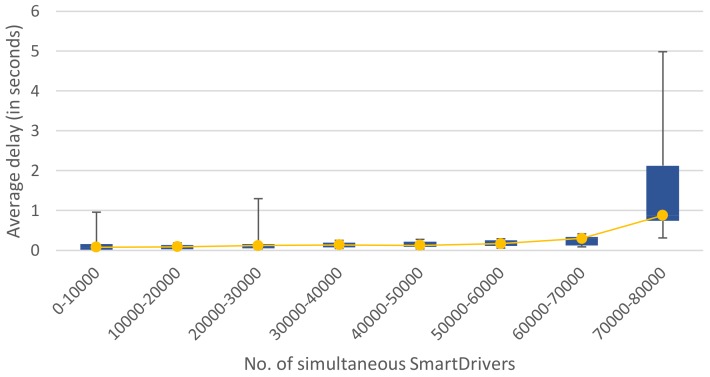
Number of simultaneous drivers that can be served and response delay for one centralised-cloud FIWARE server (see [53] for more details). The timeout threshold considered is 5 s.

**Figure 9:**
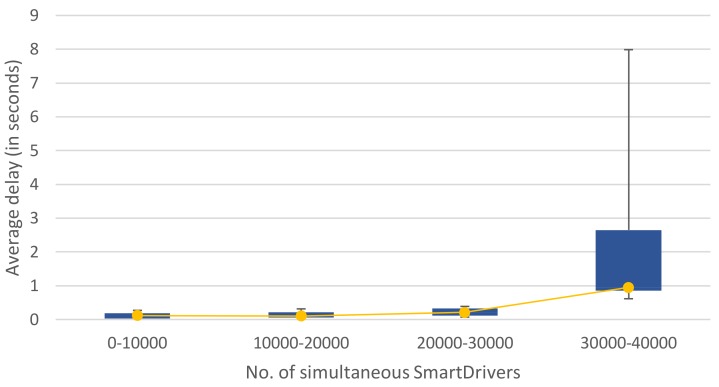
Number of simultaneous drivers that can be served and response delay for three centralised-cloud FIWARE servers (see [53] for more details). The timeout threshold considered is 5 s.

Figure 8 and Figure 9 present a summary of the performance results obtained with a cloud FIWARE cluster. Figure 8 shows that approximately 40,000 simultaneous drivers can be served optimally if one centralised-cloud server is employed, while approximately 80,000 simultaneous drivers can be served optimally when three centralised-cloud servers are employed, as shown in Figure 9. More details on the model, infrastructure and scenarios employed can be found in [53].

Finally, we can state that the behaviour and performance level of the Raspberry cluster (~32,000 active drivers) is comparable to that of one centralised-cloud FIWARE server (~40,000 active drivers) when a realistic data-intensive IoT scenario is considered.

#### 4.3.2. Storage Transfer Rate

Cloudlet computing needs to provide low latency and high bandwidth to final users when data-intensive applications are under consideration. To this aim, we specially analysed the storage transfer rate of two SBC clusters as comparison to one canonical cloud-computing cluster.

In addition to the previously explained SBC cluster setup, we configured five identical FIWARE cloud-computing virtual machines to run one instance of our simulator, each as a standard cloud-computing cluster.

To measure the storage transfer rate, we created a set of tests with the average size of vehicle location messages sent to the streaming server (approximately 220 bytes). Each simulator was configured to load 10 pre-configured paths in a range from 5 to 10 km each, increasing progressively the number of clients until a given value, logging the delay produced during the communications. All simulators were synchronised to start simultaneously, in order to achieve the peaking concurrent drivers.

Since the size of the messages sent to the streaming server was within the range of 200–240 bytes, the sequential I/O storage transfer rate of both the cloud-computing FIWARE and the SBC Raspberry Pi was measured, performing several tests of 100,000 writes and reads on each machine. The results obtained are shown in Table 4.

## 5. Infrastructure-Level Behaviour of Large-Scale Collaborative SBC-Cloudlets and Cloud Architecture Model

Once we analysed the performance results of Single Board Computer clusters in IoT scenarios at application level, we started from such performance results to model the behaviour of the proposed architecture and compared it with alternative architectures, in terms of scheduling performance, scalability, acquisition costs, and energy consumption. Such analysis and comparisons were performed to show the suitability of single-board-computer clusters as isolated and collaborative cloudlets to execute realistic IoT workflows.

**Figure 10:**
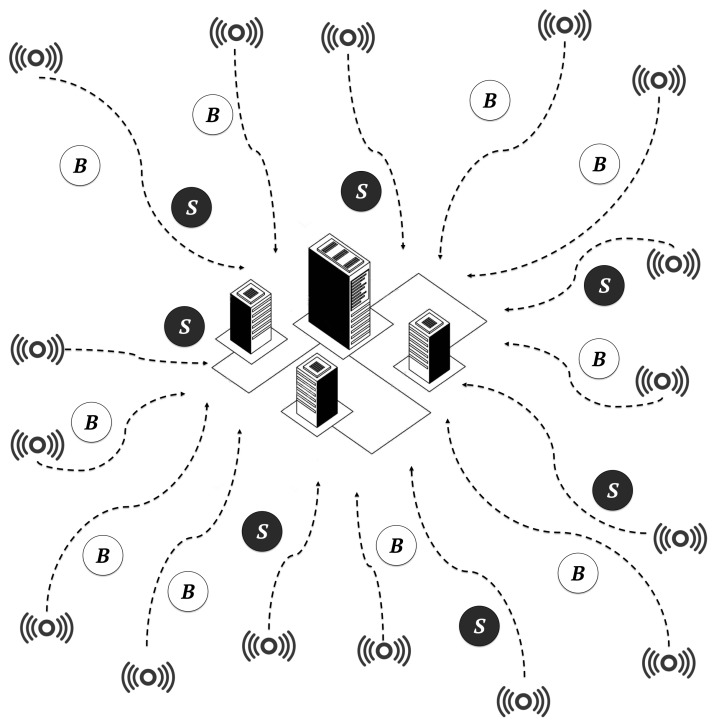
Cloud-only environment. This theoretical scenario is created only for comparison to cloudlet and cooperative environments. In this figure, a set of uniformly distributed sensors generate a workload composed of batch jobs *B* and service jobs *S*, which are deployed to a central data centre, composed of 500 high-end servers, with high latency.

**Figure 11:**
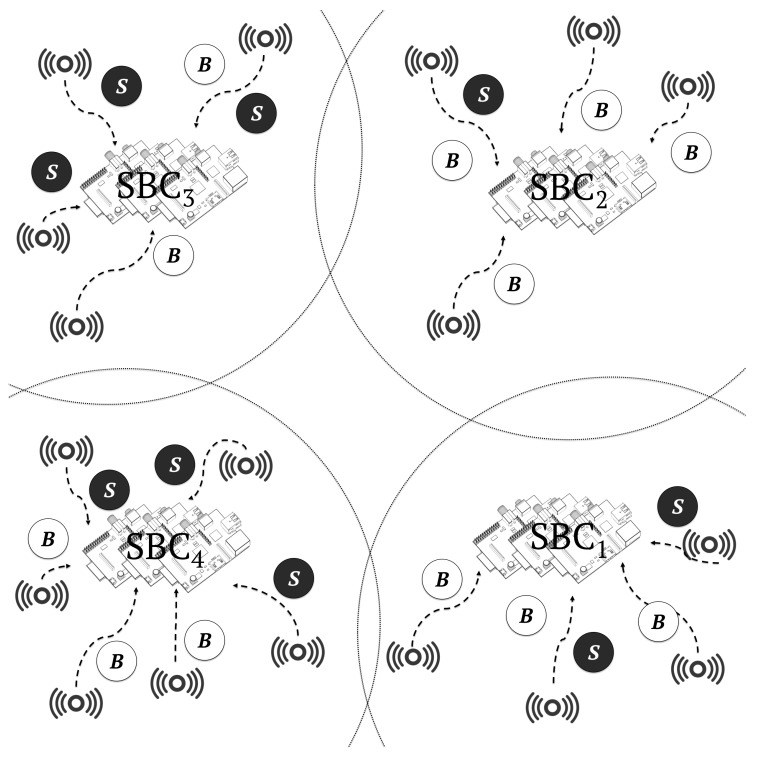
Cloudlet-only environment. This theoretical scenario is created only for comparison to cloud-computing environments. In this figure, a set of uniformly distributed sensors generate a workload composed of batch jobs *B* and service jobs *S*, which are deployed to four nearby cloudlets composed of 500 SBCs each, with low latency.

**Figure 12:**
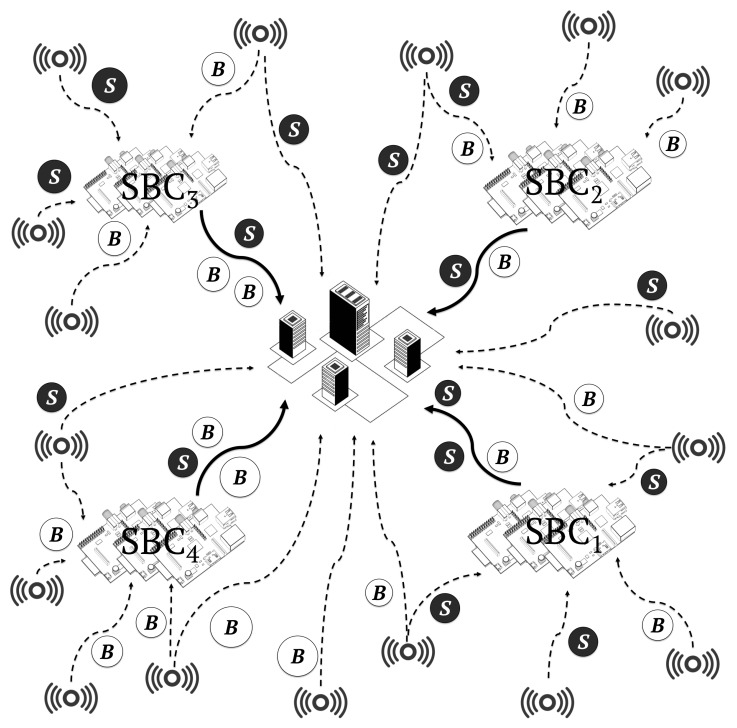
Cooperative cloud-cloudlet environment. This is our main proposed architecture model. In this figure, a set of sensors generate a workload composed of batch jobs *B* and service jobs *S*, which are deployed to both a central data centre with high latency and four nearby cloudlets composed of 500 SBC, with low latency. After the real-time processing in the cloudlets, more intensive computational tasks and data to be stored permanently are sent to the centralised cloud infrastructure, composed of 500 high-end servers, with high latency.

All simulation scenarios executed heterogeneous workloads in the form of containers, in both cloudlet and cloud infrastructures. The following four scenarios were considered:

1. In the cloud-only scenario, the entire workload was executed by a centralised cloud-Computing data centre composed of 500 high-computing-capacity servers, as shown in Figure 10. This scenario was designed to show the limitations of cloud-only architectures.
2. In the cloudlet-SBC only scenario, the entire workload was executed by four cloudlets composed of 500 SBCs each, as shown in Figure 11. This scenario was designed to show the limitations of cloudlet-only architectures.
3. In the cloudlet-CC + cloud scenario, 40% of the workload was distributed among four cloudlets composed of 60 high-end servers and 60% to a centralised data centre composed of 500 servers, respectively. This scenario showed the current less-cost-efficient cloudlet infrastructures that we aimed to improve, in terms of both costs and energy efficiency.
4. In the cloudlet-SBC + cloud scenario, 40% of the workload was distributed among four cloudlets composed of 500 SBCs and 60% to a centralised data centre composed of 500 servers, respectively. Figure 12 shows this architecture. This is the architecture model that we propose in this work.

### 5.1. Performance and Energy Models

We employed two main factors to evaluate the behaviour of the proposed models: (a) virtual machine/container queue times, which include the latency, network congestion and scheduling performance; and (b) energy consumption.

The following model was employed to compute the queue times, which are some of the key performance indicators (KPI) related to user experience:

(1) $$t_{net}^{j}=t_{lat}^{j}+t_{band}^{j}\;J=\left\{j_{1},j_{2},\ldots ,j_{n}\right\}$$

(2) $$t_{queue}^{j}=t_{net}^{j}+(t_{sch}^{j}+\sum_{c=1}^{m}t_{sch}^{cj})\;J=\left\{j_{1},j_{2},\ldots ,j_{n}\right\}\;C=\left\{c_{1},c_{2},\ldots ,c_{n}\right\}$$

where $t_{j}$ is the queue time of the job *j*. $t_{net}^{j}$ represents the network delivery time, that is, the time data require to arrive to a computing cluster. It is composed by $t_{lat}^{j}$, which denotes the latency time between the data source and the computing infrastructure, and $t_{band}^{j}$, which shows the time penalty due to bandwidth congestion. Finally, $t_{sch}^{j}+\sum_{n=1}^{m}t_{sch}^{nj}$ represents the time employed by the scheduling agents to schedule and deploy the job *j* and its tasks *N*.

The total execution time of the containers, that is, the makespan, was also computed as follows:

(3) $$t^{j}=t_{queue}^{j}+\max\left(t_{duration}^{cj}*p_{m}\right)\;M=\left\{m_{1},m_{2},\ldots ,m_{n}\right\}$$

where $t_{duration}^{cj}$ is the duration of task *c* of job *j*, and $p_{m}$ is the performance coefficient of machine *m*. The higher is the value of *m*, the more performant is machine *m*.

Regarding energy consumption, the following energy states are present for each resource: (a) *idle*: when the machine is not executing tasks; and (b) *busy*: otherwise

$t_{idle}^{i}$ denotes the period of time during which the *i*th computing resource is inactive, and $t_{busy}^{i}$ represents the period of time during which the computing resource is executing a workload. Let $P_{idle}^{i}$ and $P_{busy}^{i}$ denote the power that a particular resource needs to run in the aforementioned power states, respectively.

The time spent by a computing resource to run a task can be denoted as follows:

(4) $$t_{busy}^{ij}=\max_{t\in Tasks^{i}}C_{t}$$

$Tasks^{ij}$ being the set of tasks of the *j*th job assigned to machine $M_{i}$, whilst $C_{t}$ denotes the completion time of the *t*th task of the *j*th job.

The total time a machine is busy executing jobs and in idle state is represented as follows:

(5) $$t_{busy}^{i}=\sum_{j=1}^{j}t_{busy}^{ij}.$$

(6) $$t_{idle}^{i}=t_{total}^{i}-t_{busy}^{i}.$$

$t_{total}^{i}$ being the total execution time. Finally, we may express the energy consumption model as follows:

(7) $$\sum_{i=1}^{m}\left(P_{busy}^{i}*t_{busy}^{i}+P_{idle}^{i}*t_{idle}^{i}\right).$$

### 5.2. Simulation Tool

In this work, a simulation tool able to simulate with high performance large-scale infrastructures, usually composed of thousands of nodes, is required. Such simulation tool must provide results in terms of performance, such as job queue times and energy consumption. Several simulation tools were analysed, including: (a) edge computing simulators, such as iFogSim [56] and extensions [57,58]; and (b) cloud computing simulators, such as CloudSim [59], CloudSched [60], GreenCloud [60], and SCORE [61].

CloudSim and edge/fog Computing simulators, as they are based on CloudSim, present serious limitations in terms of performance when large-scale infrastructures are considered [62,63]. Other simulation tools, such as CloudSched or GreenCloud, only focus on networking subsystems, providing insufficient results to evaluate such a complex large-scale infrastructure.

In this work, we extended the previously developed SCORE simulation tool [61] to support cloudlet infrastructures. This simulator allows the simulation of large-scale infrastructures in a performant way, while providing flexibility for the usage of various resource managing models, scheduling algorithms and energy-efficiency policies.

### 5.3. Workload

In this paper, we follow the literature trends presented in [64]. In the aforementioned work, IDC estimates that approximately 40% of data will be analysed on edge nodes and clusters. Synthetic workloads following these industry trends were generated for each simulation run (a deeper explanation can be found in [65]). The workload considered in this work was composed of two types of jobs:

(a) Batch jobs perform a computing-intensive workload and then finish. MapReduce jobs are an example of a *Batch* job.
(b) Service jobs represent long-running jobs with no determined end. Long-running services, such as databases, streaming engines, and web servers, are representative Service jobs.

In addition, two kinds of different workloads were considered:

(a) Data-intensive workloads consist of jobs composed of tasks that lightly process large amounts of data. Batch jobs in this workload require large amounts of bandwidth and data, but low computing capacity.
(b) Compute-intensive workload have jobs composed of tasks that perform complex computations. Batch jobs in this workload require high computing capacity, while they do not require long data transmissions.

The following job attributes were considered in this study for both kind of workloads: (a) inter-arrival time, representing the time elapsed between two consecutive jobs; (b) number of tasks jobs are composed of; (c) duration; and (d) CPU and RAM utilisation.

All attributes were generated by the means of the exponential distribution, which is the standard statistical function to model the arrival of jobs to data centres.

### 5.4. Experimentation Results

**Figure 13:**
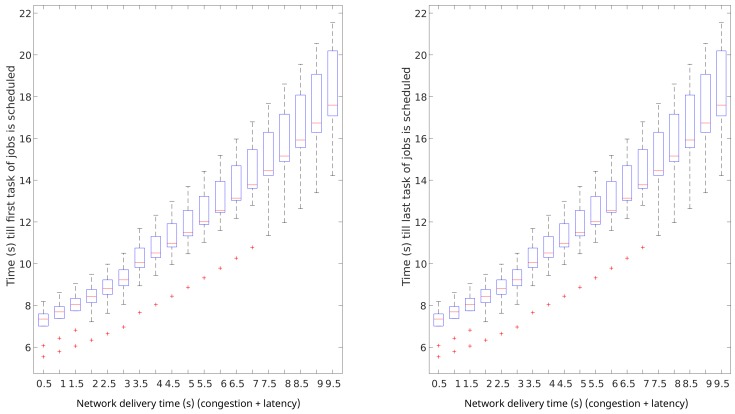
Network impact on queue times in centralised cloud-computing data centres.

**Figure 14:**
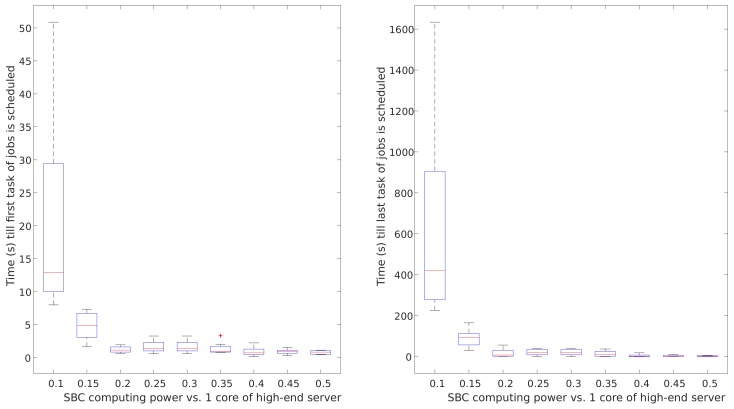
SBC computing power impact on queue times in distributed cloudlet computing SBC clusters.

In this section, we show the experimental results of the aforementioned four scenarios. First, we characterise the impact in terms of performance (queue times) of two main considered parameters:

- Network delivery time $t_{net}^{j}$ impacts heavily on the makespan of data-intensive workloads, and has a minor impact in terms of scheduling performance. The scheduling performance is represented by the job queue times. The results of this analysis are graphically shown in Figure 13. In this figure, the impact of a range of values for $t_{net}^{j}$ on queue times are presented. It can be noticed that the network delivery time impacts negatively in terms of queue times linearly. Furthermore, when very high values are present, $t_{net}^{j}$ makes the system performance more unpredictable.
- Impact of the computing power of SBCs on queue times in SBC clusters was compared to the computing power of one core in typical cloud-computing servers. The results of this analysis are graphically shown in Figure 14. It can be noticed that the computing capacity is the key factor to achieve short queue times in prototypical IoT scenarios if compute-intensive workloads are under consideration. This analysis shows that SBCs may provide predictable performance results if the computing capacity of the SBC raises to 30% of that of one core of current typical cloud-computing servers. With the required improvements, SBCs can become an optimal candidate for edge-computing nodes.

We followed the trends shown in the previous analysis to parameterise each simulation scenario presented in Table 5 and Table 6. Even though in this section we try to consider the future SBC trends instead of simulating one concrete Single-Board-Computer configuration, we used the Odroid performance parameters as representative for near-future trends of performance and energy consumption for SBCs as to provide a realistic baseline for researchers who want to deploy their own SBC clusters based on the research results. This assumption was confirmed by the characteristics of the Raspberry Pi, which were presented a short time after performing the experimentation of this section. Therefore, the following realistic parameters were considered:

- $t_{sch}^{j}$ was set to 150 ms and $\sum_{c=1}^{m}t_{sch}^{cj}$ to 100 ms. A range of values for $t_{net}^{j}$ was considered for cloud facilities, while $t_{net}^{j}$ took a value of 10 ms for cloudlet clusters.
- The computing power of SBCs was set to 20% of one core of a cloud server.
- The number and duration of tasks were similar for both experiment sets, while $p_{m}$ was set to 1 for cloud servers, and 0.2 for Single-Board Computers.
- $P_{busy}^{i}$ was set to 110 W and $P_{idle}^{i}$ to 50 W per cloud CPU. respectively. $P_{busy}^{i}$ was set to 15 W and $P_{idle}^{i}$ to 8 W per SBC.

Approximately 6500 Batch and 600 Service jobs were executed during seven days for each simulation run. Thus, in the cloudlet-only scenario, four clusters composed of 500 SBCs each executed this workload, while in the cloud-only scenario 500 high-end (HE) servers were considered. Sixty high-end servers were used in each cloudlet cluster in Scenario 3. Both cloud and cloudlet clusters employed a centralised monolithic scheduler and started the operation time with 20% of resource utilisation, following the trends present in industry [66].

Each simulation was run five times during seven days of operation time, and an ANOVA test with trust level p<0.05 was performed. The average values for all KPIs are shown in Table 4 and Table 5 for data and compute-intensive environments, respectively.

These results show that the network delay has a minor impact in terms of scheduling performance. Even in the worst-case scenario for centralised cloud-computing infrastructures, that is, the data-intensive workload, high latency times only increased approximately 10% the queue times. It can be noticed that federated cloudlets could alleviate the scheduling congestion of centralised infrastructures. This behaviour is related to the monolithic centralised resource-managing strategy, and a deep analysis should be performed in the future with other resource-managing models, such as the two-level and shared-state centralised models, or even distributed and hybrid models.

It is also clearly shown that centralised clouds had more than sufficient computing power to execute the workloads, since they could schedule all the tasks in a job in only one scheduling operation. Thus, this scenario showed similar $t_{j}^{first}$ and $t_{j}^{full}$ results, which represent the time until the first and last task of a job are scheduled, respectively.

In data-intensive environments, isolated clouds suffer from both scheduling congestion, due to the centralised scheduling model, and network delay. In scenarios of high latency, the completion time (makespan) of batch jobs increased 50% compared to that of low-latency environments, and 60% compared to that of isolated cloudlet architectures. Service jobs were not affected, since they had no determined end (they were killed when no longer needed).

The theoretical isolated SBC-cloudlets architecture would provide a good result in terms of scheduling performance, therefore reducing the queue times by a factor of 7×, approximately, if enough storage capacity were provided by these clusters. This behaviour is due to more less-powerful cores are provided by this infrastructure. It is also worth mentioning that, even though the acquisition costs and energy consumption of SBC-cloudlets were approximately nine and five times lower, respectively, high-end (HE)-cloudlets provide similar results to those of SBC-cloudlets, except for the makespan, which presented a 10% reduction compared to that of SBC-cloudlets.

For realistic collaborative cloud + cloudlet scenarios, the results provided by both the SBC and the HE-cloudlets are similar, even if the acquisition cost of the SBC scenario was approximately 30% lower. Both scenarios presented queue times approximately three times shorter than isolated clouds and achieved 10% reductions in terms of makespan. The network delay had a minor impact in the centralised clouds for these scenarios, since data-intensive tasks were pre-processed by the closest cloudlets.

On the other hand, when compute-intensive scenarios were considered, SBC cloudlets only improved the initial scheduling performance $t_{j}^{first}$, since they alleviated the centralised scheduler congestion. However, due to the low computing power, they could not to provide fast scheduling decisions to all tasks in a job, as can be noticed in the $t_{j}^{full}$ and makespan results, which are approximately 12 and 6 times longer than those of cloud-only architectures, respectively. Therefore, due to the results shown in Figure 13, SBCs do not provide sufficient computing power to execute all the workload by themselves. Such a behaviour may be also noticed in the high resource consumption results. However, the low cost ($70,000 vs. $1,200,000), and the low energy consumption (~10%) of this scenario compared to the cloud-only scenario must be noticed.

Collaborative cloudlet + cloud architectures present different behaviours in compute-intensive environments. The SBC-cloudlets could compete with HE-cloudlets only in terms of queue time until the first task of a job was scheduled $t_{j}^{first}$. This is due to the higher number of low-computing-capacity cores. Notwithstanding, the low computing capacity of SBCs penalised heavily jobs with high computing requirements (batch jobs) when the time until the last task of a job $t_{j}^{full}$ and the makespan were analysed, increasing by four and three times the results provided by HE-cloudlets, respectively.

The results clearly show that the collaborative HE-cloudlets + cloud system provides better results for all KPIs, and is preferred if no cost limitation were present. On the other hand, the collaboration between SBC-cloudlets and centralised cloud infrastructures may be a cost-effective alternative for latency-sensitive, data-intensive, and low-compute scenarios.

## 6. Conclusions and Future Work

In this paper, the following contributions have been presented:

- Cost and resource-efficient cloudlet Computing architecture model based on single-board-computer clusters.
- Analysis on single board computers, such as Raspberry Pi 3, can successfully execute real-time realistic IoT workloads, such as the presented traffic-system scenario.
- Evaluation of results in terms of performance, energy consumption and acquisition costs of the proposed model compared to the architectures found in the literature, both for data and compute-intensive scenarios.

As stated throughout the paper, a cluster made of SBCs is a small- and medium-scale cost-effective solution when latency-sensitive, data-intensive and low-computing scenarios are considered. These single board computers are also easy to assemble and configure, therefore achieving highly versatile systems.

It has been shown that the proposed architecture model based on SBC clusters can catch up with high-end micro data centres in realistic scenarios, when workloads that require low computing power are considered. Good performance levels can be achieved while keeping operational costs low, saving vast amounts of energy, and the carbon dioxide related to energy consumption. In addition, as we show in the paper, it is expected that SBCs improve the performance levels for computing-demanding workloads as long as their computing capacity increases.

As future work, we intend to extend the present work by performing:

- Validation of the obtained results in a real infrastructure.
- Development of full architecture cost model, including operations, maintenance and network costs.
- Improvements on the simulation tool to consider more complex networking topologies.
- Improvements on the simulation tool to consider more complex scheduling algorithms between cooperative cloudlets and cloud-computing infrastructures.
- Analysis on the impact of resource-managing frameworks in the proposed model.
- Analysis on the impact of energy-efficiency policies in the proposed model.
- Evaluation of cost models for the proposed architecture using productive analysis models, such as Data Envelopment Analysis [67].

## Figures and Tables

**Table 1:** Features summary for the edge-computing paradigms analysed. In this paper, we propose a model for the improvement of cost and energy consumption of cloudlet architectures.

Feature	Multi-Access Edge	Fog	Cloudlet
Cloud-providers integration	Medium	Medium	Easy
Distance	Close	Close	Close
Availability	Average	High	High
Latency	Low	Low	Low
Acquisition Cost	Medium	Low	High
Energy consumption	Low	Low	Medium
Reliability	Medium	High	Medium

**Table 2:** Single-Board-Computer micro data-center performance results without compression vs. using compression.

Measure	Without Compression	LZ4 Compression
Avg. network upload bandwidth	8.7 MB/s	8.6 MB/s
Avg. network download bandwidth	8.1 MB/s	4.4 MB/s
Avg. CPU usage	38.2%	42.7%
Avg. memory usage	90.1%	91.3%
Avg. swap usage	42.6%	40.5%
Avg. core temperature	56.2 °C	57.8 °C

**Table 3:** Number of simultaneous users tests.

Simultaneous Drivers	Highest Slow Responses Rate	Highest Delay
10,000	0%	0.6 s
20,000	3%	0.7 s
30,000	18%	0.9 s
35,000	47%	1.6 s
40,000	77%	1.8 s
60,000	95%	2.7 s
80,000	96%	4.1 s

**Table 4:** Storage average transfer rate using a message size of 220 bytes.

	Raspberry Pi 2	Raspberry Pi 3	FIWARE
Max	4.8 MB/s	9.7 MB/s	69.6 MB/s
Min	3.0 MB/s	7.2 MB/s	54.8 MB/s
Avg	4.0 MB/s	5.4 MB/s	61.4 MB/s

**Table 5:** Performance, energy consumption and costs results for each scenario for data-intensive workloads. $t_{j}^{net}$ represents the network delay, while $t_{j}^{first}$ and $t_{j}^{full}$ represent the time a job spends in queue until its first task and its last task is scheduled, respectively. B means batch jobs while S means service jobs. To compute acquisition costs, we took average Dell PowerEdge R640 server and Raspberry Pi 3 prices, 2500 and 35 dollars, respectively.

Scenario	$t_{j}^{\mathrm{net}}$	Resource	$t_{j}^{\mathrm{first}}$ (s)		$t_{j}^{\mathrm{full}}$ (s)		Makespan (s)		MWh	Cost
	(ms)	Occ. (%)	B	S	B	S	B	S	cons.	(M$)
Isolated										
Cloud	50	20.80	5.70	5.56	5.70	5.56	99.21	2003.19	9.73	1.20
Cloud	150	21.00	5.61	5.33	5.61	5.33	114.66	1985.85	9.75	1.20
Cloud	300	21.11	6.26	5.33	6.26	5.33	142.27	1980.65	9.76	1.20
HE-Cloudlets	10	22.46	0.91	0.77	3.21	2.46	88.24	2058.18	4.77	0.60
SBC-Cloudlets	10	18.26	0.87	0.77	1.85	1.46	95.90	1996.77	1.03	0.07
Collaborative										
Cloud +	50	19.18	1.71	1.38	2.10	1.82	90.62	1979.64	14.47	1.80
HE-Cloudlets	10	17.52								
Cloud +	150	19.22	1.72	1.39	2.11	1.59	90.62	1979.64	14.48	1.80
HE-Cloudlets	10	17.70								
Cloud +	300	19.18	1.73	1.41	2.12	1.60	90.62	1979.64	14.48	1.80
HE-Cloudlets	10	18.45								
Cloud +	50	19.24	1.69	1.46	1.74	1.95	93.62	2019.96	10.72	1.27
SBC-Cloudlets	10	14.11								
Cloud +	150	19.19	1.70	1.47	1.75	1.72	93.62	2019.96	10.72	1.27
SBC-Cloudlets	10	14.68								
Cloud	300	19.23	1.72	1.49	1.76	1.74	93.62	2019.96	10.73	1.27
SBC-Cloudlets	10	15.13								

**Table 6:** Performance, energy consumption and costs results for each scenario for compute-intensive workloads. $t_{j}^{net}$ represents the network delay, while $t_{j}^{first}$ and $t_{j}^{full}$ represent the time a job spends in queue until its first task and its last task is scheduled, respectively. B means batch jobs while S means service jobs. To compute acquisition costs, we took average Dell PowerEdge R640 server and Raspberry Pi 3 prices, 2500 and 35 dollars, respectively.

Scenario	$t_{j}^{\mathrm{net}}$	Resource	$t_{j}^{\mathrm{first}}$ (s)		$t_{j}^{\mathrm{full}}$ (s)		Makespan (s)		MWh	Cost
	(ms)	occ. (%)	B	S	B	S	B	S	cons.	(M$)
Isolated										
Cloud	50	20.82	5.19	5.18	5.19	5.18	98.61	2008,41	9.72	1.20
Cloud	150	20.82	5.23	5.22	5.23	5.22	98.61	2008.41	9.72	1.20
Cloud	300	20.82	5.29	5.28	5.29	5.28	98.60	2008.41	9.72	1.20
HE-Cloudlets	10	22.32	0.88	0.85	2.96	3.32	91.56	2051.33	4.73	0.60
SBC-Cloudlets	10	34.37	3.02	3.23	62.05	69.94	575.97	2066.43	1.67	0.07
Collaborative										
Cloud +	50	19.21	1.75	1.42	2.09	1.64	90.62	1979.64	14.47	1.80
HE-Cloudlets	10	17.42								
Cloud +	150	19.27	1.76	1.43	2.10	1.65	90.62	1979.64	14.48	1.80
HE-Cloudlets	10	17.65								
Cloud +	300	19.28	1.78	1.44	2.11	1.67	90.62	1979.64	14.48	1.80
HE-Cloudlets	10	18.07								
Cloud +	50	19.24	1.81	1.43	6.41	6.37	241.46	2002.00	10.84	1.27
SBC-Cloudlets	10	21.32								
Cloud +	150	19.29	1.82	1.44	6.42	6.38	241.45	2002.00	10.84	1.27
SBC-Cloudlets	10	21.34								
Cloud	300	19.34	1.84	1.46	6.44	6.39	241.46	2002.00	10.85	1.27
SBC-Cloudlets	10	21.42								
